# The host ubiquitin-dependent segregase VCP/p97 is required for the onset of human cytomegalovirus replication

**DOI:** 10.1371/journal.ppat.1006329

**Published:** 2017-05-11

**Authors:** Yao-Tang Lin, James Prendergast, Finn Grey

**Affiliations:** Division of Infection and Immunity, The Roslin Institute, University of Edinburgh, Easter Bush, Midlothian, United Kingdom; University of Arizona, UNITED STATES

## Abstract

The human cytomegalovirus major immediate early proteins IE1 and IE2 are critical drivers of virus replication and are considered pivotal in determining the balance between productive and latent infection. IE1 and IE2 are derived from the same primary transcript by alternative splicing and regulation of their expression likely involves a complex interplay between cellular and viral factors. Here we show that knockdown of the host ubiquitin-dependent segregase VCP/p97, results in loss of IE2 expression, subsequent suppression of early and late gene expression and, ultimately, failure in virus replication. RNAseq analysis showed increased levels of IE1 splicing, with a corresponding decrease in IE2 splicing following VCP knockdown. Global analysis of viral transcription showed the expression of a subset of viral genes is not reduced despite the loss of IE2 expression, including UL112/113. Furthermore, Immunofluorescence studies demonstrated that VCP strongly colocalised with the viral replication compartments in the nucleus. Finally, we show that NMS-873, a small molecule inhibitor of VCP, is a potent HCMV antiviral with potential as a novel host targeting therapeutic for HCMV infection.

## Author summary

Viruses are obligate intracellular pathogens, meaning that they are completely dependent on the host cellular machinery to replicate. Identifying which host genes are necessary for virus replication extends our understanding of how viruses replicate, how cells function and provides potential targets for novel antivirals. Here, we show that a cellular factor called valosin containing protein (VCP) is essential for human cytomegalovirus replication. We demonstrate that VCP is required for the expression of an essential virus gene called IE2. Finally we show that a chemical inhibitor of VCP is a potent antiviral against human cytomegalovirus, demonstrating the potential for VCP inhibitors as novel therapeutics against this virus.

## Introduction

Human cytomegalovirus (HCMV) is a highly prevalent herpesvirus, infecting 30 to 100% of the global population depending on the socio-economic status. Although normally asymptomatic in healthy individuals, HCMV infection is a significant cause of morbidity and mortality in immunocompromised populations, individuals with heart disease and recipients of solid organ and bone marrow transplant. HCMV is also the leading cause of infectious congenital birth defects [1–9].

**Fig 1:**
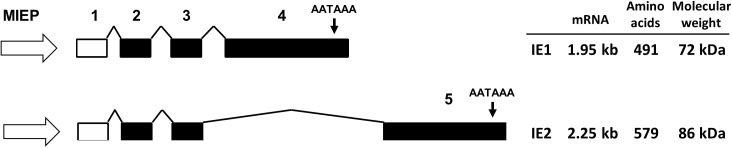
Schematic representation of differential splicing of IE1 and IE2. IE1 and IE2 are derived from the same primary transcript, driven by the major immediate early promoter. Differential splicing and polyadenylation of the terminal exon dictates expression of IE1 or IE2. Filled boxes indicate coding exons whereas the open box represents a non-coding exon.

During infection, HCMV initiates a programmed cascade of gene expression, resulting in production of infectious virus. Two of the first genes to be expressed are the major immediate early (MIE) genes IE1 (IE72) and IE2 (IE86). The MIE proteins have multiple roles during infection including transactivation of viral genes, which drives replication and virus production [10–12]. Because of this, they are thought to play a pivotal role in controlling the switch between latent and productive infection in HCMV [13,14]. While IE1 is required for efficient virus replication at low multiplicity of infection [13,14], IE2 expression is essential, with deletion resulting in non-viable virus [15]. IE1 and IE2 are generated from the same primary transcript by differential splicing and alternative polyadenylation [10,12,16]. They share the first three exons, with splicing to the fourth or fifth exon determining expression of IE1 or IE2 transcript, respectively (Fig 1). Independent polyadenylation signals exist downstream of both exon four and exon five. Such genomic arrangements, that require terminal exon skipping, are considered relatively unusual in the host cell, with specific factors and mechanisms involved in regulating the process not fully understood [17].

Valosin containing protein (VCP) belongs to the hexameric AAA ATPase family and plays a pivotal role in ubiquitin mediated signaling through remodeling target proteins, often leading to proteosomal degradation [18]. VCP contains two ATPase domains, which hydrolyze ATP to generate the energy required to remodel or unfold target proteins. Through this action, VCP is able to segregate target proteins from associated cellular membranes or larger protein complexes. Once segregated, the target protein is relocalised or degraded via the proteosomal complex. VCP can also affect which proteins are modified through its interaction with multiple ubiquitin regulatory co-factors, making VCP a central signalling hub for ubiquitin mediated regulation. In addition to ubiquitin, VCP has been linked to other post-translational modifiers such as SUMO and Nedd8 [19,20]. As such it is linked to a wide range of biological functions, including protein quality control, autophagy, chromatin remodeling, DNA damage response and more recently RNA processing [21]. Functionally, VCP plays a central role in protein homeostasis by facilitating proteosomal degradation of misfolded or damaged proteins as part of the endoplasmic reticulum associated degradation pathway (ERAD). In addition to its role in protein degradation, VCP has been linked to non-degradative functions involving removal and relocation of proteins from membranes and protein complexes. Examples include removal of Aurora B protein from mitotic chromosomes, removal of transcription factors from chromatin and disassembly of RNP complexes [22,23]. It has also been linked to a number of functional roles in virus replication including entry of Sindbis virus and replication of poliovirus [24–26]. While VCP plays a role in US11-specific degradation of MHC class I protein during HCMV infection [27] a direct role for VCP in the replication of HCMV has not previously been reported.

Using a focused siRNA screen, we identified VCP as an essential factor for HCMV replication. We show that VCP is essential for the onset of virus replication and knockdown of VCP results in changes in alternative splicing of the MIE transcripts, loss of IE2 expression and ultimately failure in virus replication.

## Results

### VCP is an essential host factor for HCMV replication

**Fig 2:**
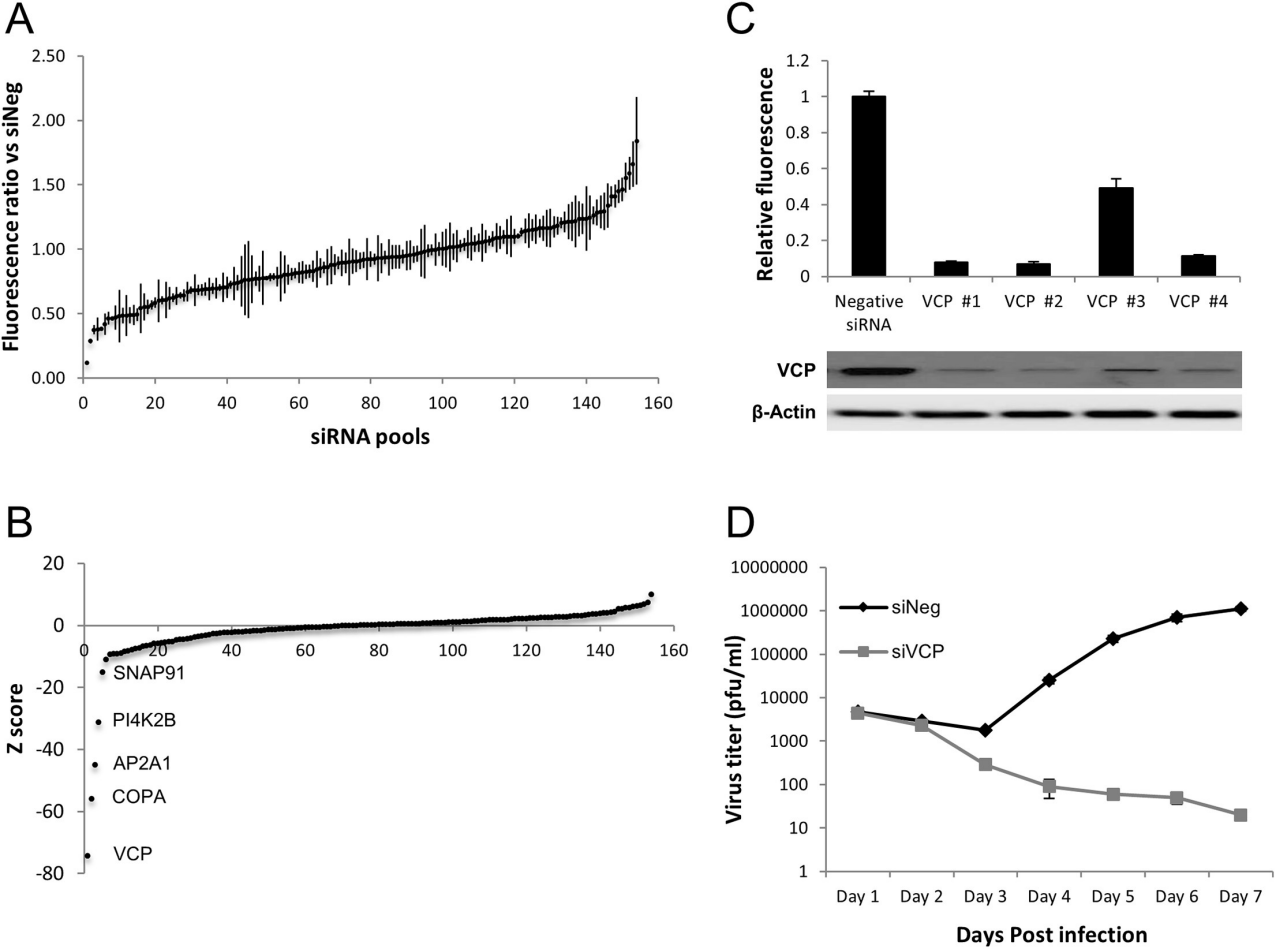
Knockdown of the cellular gene VCP results in a complete block in HCMV replication. Human primary fibroblast cells were transfected with 160 siRNA pools against membrane organization genes then infected with a GFP expressing HCMV virus. (A) Relative GFP fluorescence levels compared to cells transfected with a negative control siRNA for all 160 siRNA pools. Assays were performed in duplicate with three biological repeats with standard deviations shown. (B) GFP levels ranked by Z-score. (C) VCP deconvoluted siRNAs were tested for VCP knockdown by western blot and virus replication by relative GFP fluorescence to negative control siRNA transfected cells. GFP levels were measured 144 HPI and assays performed in duplicate. (D) HCMV replication was measured by plaque analysis following transfection with VCP siRNA or negative control siRNA. Cells were infected at an MOI of three and performed in duplicate with two biological repeats.

Previously, we identified the host gene ATP6V0C as a critical factor in HCMV virus production [28]. ATP6V0C is a component of the vacuolar ATPase, and among other functions, is involved in membrane organization [29]. To identify additional host membrane organization factors involved in HCMV replication we performed a focused siRNA screen against 160 host genes involved in membrane organization using pools of four siRNAs against each target. Primary human fibroblast cells were double transfected with each siRNA pool in 96 well format. Two days post-transfection, the cells were infected with a low passage HCMV strain, TB40E, expressing GFP from an SV40 promoter. GFP levels were monitored over a period of seven days by fluorometry to determine virus infection and replication levels (S1 Table). Analysis of the screen indicated both increased and decreased virus replication ranging from 88% reduction in GFP levels to a 84% increase (Fig 2A). Z-score analysis based on three biological repeats of the siRNA screen identified five clear outliers that resulted in reproducible reductions in virus replication, as measured by GFP fluorescence intensity (Fig 2B). Of these, knockdown of VCP resulted in the largest negative Z-score. To validate that the observed phenotype was due to knockdown of VCP, rather than potential off-target effects, the original siRNA pool was deconvoluted to test the four individual siRNAs against VCP. All four siRNAs resulted in reduced VCP expression and reduced HCMV replication based on GFP fluorescence (Fig 2C). The third siRNA of the pool generated less efficient VCP knockdown, and corresponded with less inhibition of HCMV in the siRNA assay, providing additional evidence that knockdown of VCP results in a direct reduction in virus replication. These results indicate that the initial observed phenotype is due to specific knockdown of VCP rather than off-target artefactual effects of the siRNAs. Although GFP expression serves as an effective read-out for virus replication, loss of GFP signal may not necessarily reflect reduced virus production. To determine the direct effect of VCP knockdown on HCMV virus production, one-step growth curves were generated. Primary human fibroblast cells were transfected with siRNA pools targeting VCP or a negative control non-targeting siRNA. Two days post transfection cells were infected at a multiplicity of infection (MOI) of three with TB40E-GFP. Supernatant was collected at 24-hour time points for seven days total. Knockdown of VCP resulted in significant reduction in HCMV replication, with viral titers over four log_10_ lower in cells depleted of VCP compared to negative control cells at day seven (Fig 2D). No amplification of infectious virus was observed in cells knocked down for VCP, suggesting a complete block in virus replication. Cell viability assays showed a moderate decrease in cell viability at 5 days post transfection with VCP siRNA compared to negative control siRNA, although visual inspection of cells did not indicate gross cytotoxicity and the reduction is not sufficient to account for the substantial reduction in virus replication (S1 Fig).

### VCP is required for IE2 expression but not IE1 expression

**Fig 3:**
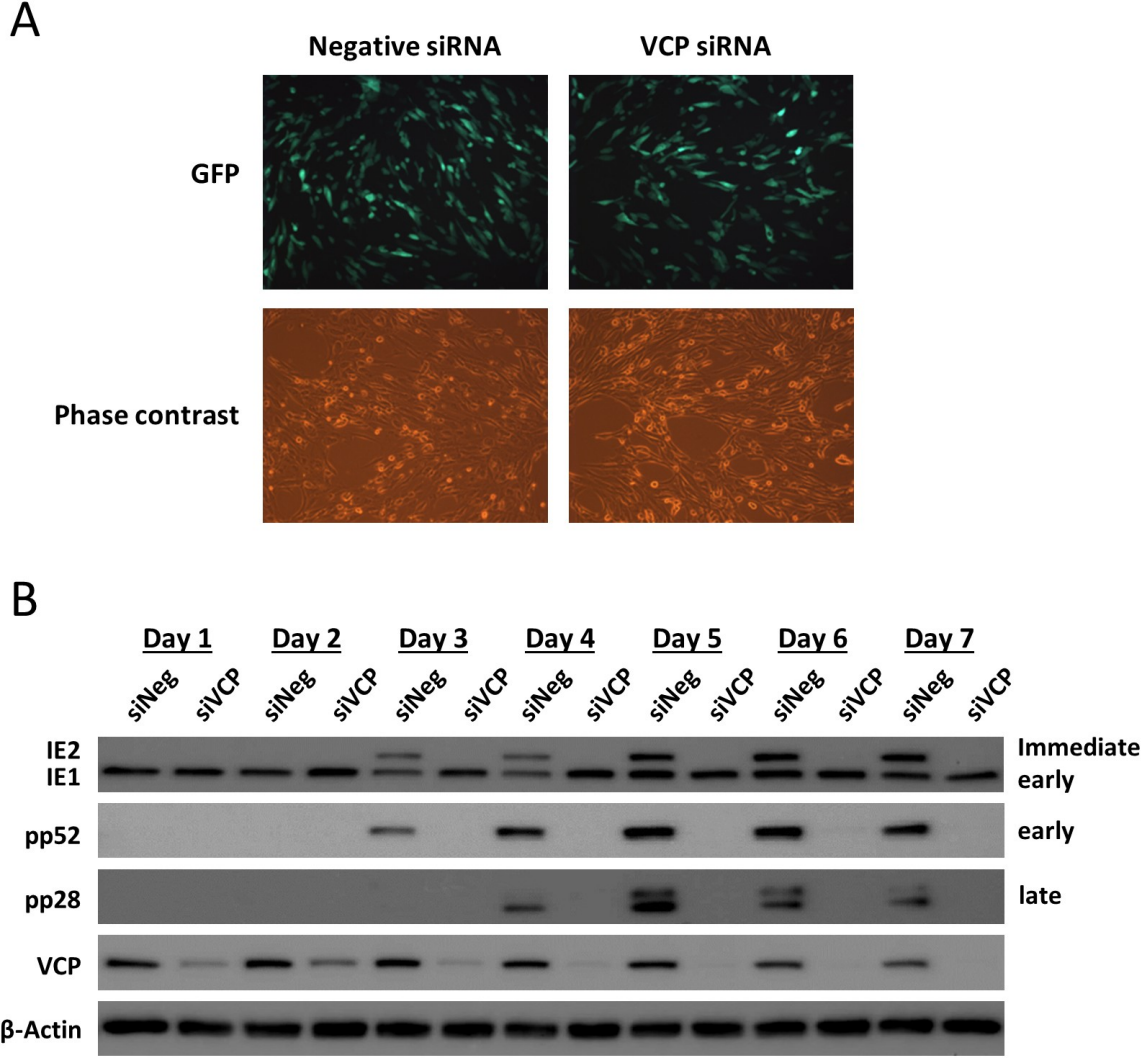
Knockdown of VCP results in a specific loss of the major immediate early protein IE2. (A) Fluorescence microscopy demonstrates that all cells are infected with HCMV at 48 HPI following transfection with VCP siRNA, indicating no major effect on virus entry and translocation to the nucleus. (B) Western blot analysis for viral proteins of each major kinetic class shows a clear loss of IE2 expression and downstream early and late viral proteins.

Further characterization was performed to determine at what stage of the HCMV replication cycle VCP is required. Analysis of GFP fluorescence at 48 hours post infection (HPI) indicated that all cells were infected, demonstrating that VCP expression is not required for virus entry, translocation of genome to the nucleus or initial transcription of the viral genome (Fig 3A). Western blot analysis was performed to measure the levels of each of the major kinetic classes of HCMV genes, including immediate early (IE1 and IE2), early (pp52) and late (pp28), to define when disruption in virus replication occurs (Fig 3B). In control cells, protein expression of each class of virus gene is clearly observed. However, in cells knocked down for VCP, neither IE2 nor downstream viral gene expression was detected. Strikingly, despite being encoded by the same primary transcript, IE1 expression levels were higher relative to control cells, up until day four post infection. This data indicates that VCP expression is required for the onset of HCMV replication and suggests knockdown of VCP affects expression of the major immediate early genes.

### Knockdown of VCP does not result in IE2 protein destabilisation

**Fig 4:**
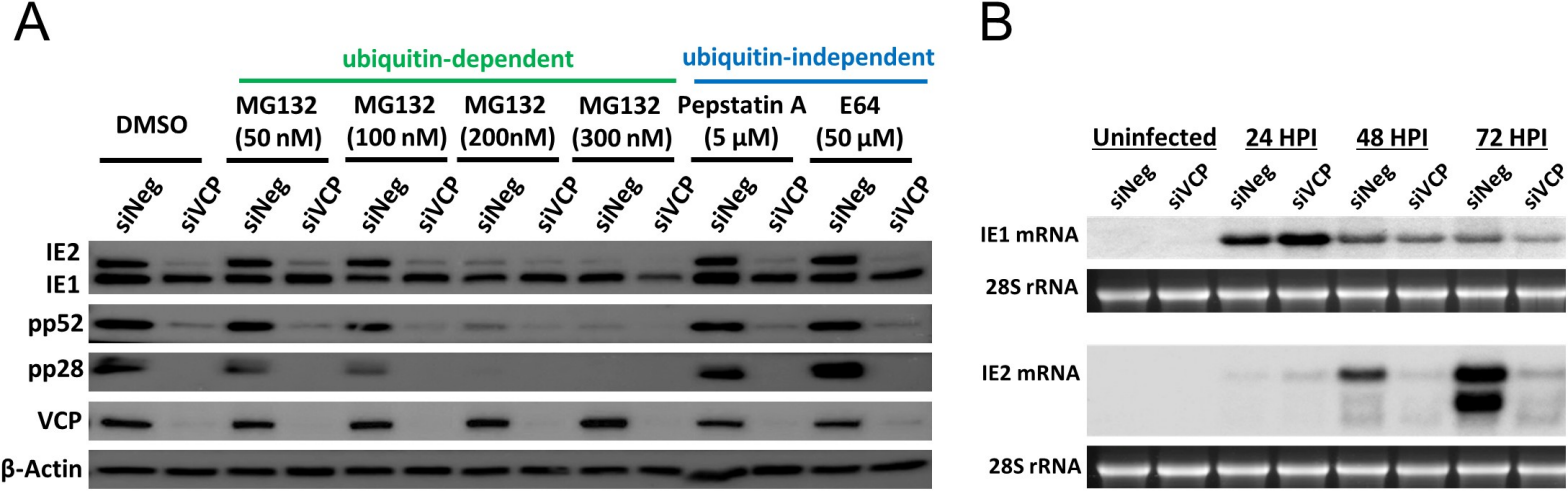
Knockdown of VCP results in substantial reduction in IE2 transcript. (A) Following transfection with VCP siRNA or negative control siRNA, cells were treated with proteasome or protease inhibitors, then infected with HCMV. Viral protein levels were measured by western blot analysis. Inhibition of protein degradation did not rescue IE2 expression in VCP knockdown cells. (B) Fibroblast cells were transfected with VCP or negative control siRNA and infected with HCMV. Total RNA was harvested at the indicated time points and IE1 and IE2 transcript levels determined by Northern blot analysis. 28S rRNA stained with ethidium bromide is shown as loading control.

Because knockdown of VCP resulted in specific loss of IE2 but not IE1, we hypothesized that VCP may play a role in IE2 protein stability, transcript stability or regulation of alternative splicing and polyadenylation. To determine whether VCP was required for IE2 protein stability we inhibited the two main pathways of protein degradation using MG132 (proteasome inhibitor–ubiquitin dependent) and pepstatin A or E64 (protease inhibitors–ubiquitin independent) in the context of VCP knockdown (Fig 4A). If VCP regulation of IE2 was due to protein degradation, inhibition of these pathways should rescue IE2 protein levels. Neither MG132, pepstatin A, nor E64 rescued the loss of IE2 proteins levels following knockdown of VCP. Instead, higher concentrations of MG132 phenocopied VCP knockdown in control cells, indicating that proteosomal function is required for IE2 but not IE1 gene expression. As VCP targets ubiquitinated proteins for proteosomal degradation, this result suggests that an intermediate protein, targeted by VCP, may inhibit IE2 expression and is consistent with previous publications showing inhibition of the proteasome reducing IE2 expression and HCMV replication [30].

### Knockdown of VCP results in loss of IE2 transcript expression

To determine whether regulation of the MIE genes was occurring at the RNA level, Northern blot analysis was performed. Fibroblast cells transfected with siRNA against VCP or control siRNA were infected 48h post transfection at high MOI with TB40E and total RNA harvested at 24, 48 and 72 HPI. As shown in Fig 4B, knockdown of VCP resulted in a substantial reduction of IE2 RNA levels at 48 and 72 HPI, including the smaller IE60 and IE40 species. This result was confirmed by qRT-PCR, using primers specific for IE1 and IE2 transcript. Supplemental Fig 2A demonstrates the clear difference in the kinetics of IE1 and IE2 transcript accumulation in control cells, with IE1 transcript levels rapidly increasing following infection, while IE2 levels initially increase, then plateau between 12 and 24 HPI, before accumulating to substantially higher levels from 24 HPI onwards. This accumulation fails to occur following knockdown of VCP, where levels of IE2 do not substantially increase after 24 HPI, in contrast to control cells (S2B and S2D Fig). MIE transcription is also affected at earlier time points with levels of both IE1 and IE2 reduced at 6 HPI and increased at 24 HPI (S2C and S2D Fig). An increase in IE1 and IE2 RNA levels at 24 HPI may be a result of increased MIE promoter activity due to the associated loss of IE2 protein expression at early time points, as IE2 is a negative regulator of the MIE promoter. IE1 transcript levels decrease rapidly 24 to 48 HPI, while protein levels remain high, suggesting that IE1 protein is stable and the increase in IE1 transcription at 24 hours may contribute to the prolonged increase in IE1 protein levels. These results suggest that VCP may be affecting other aspects of MIE transcription. However, Northern, Western and qRT-PCR data are all consistent with a substantial effect on IE2 expression from 24 HPI onwards.

### Knockdown of VCP does not reduce IE2 transcript stability

**Fig 5:**
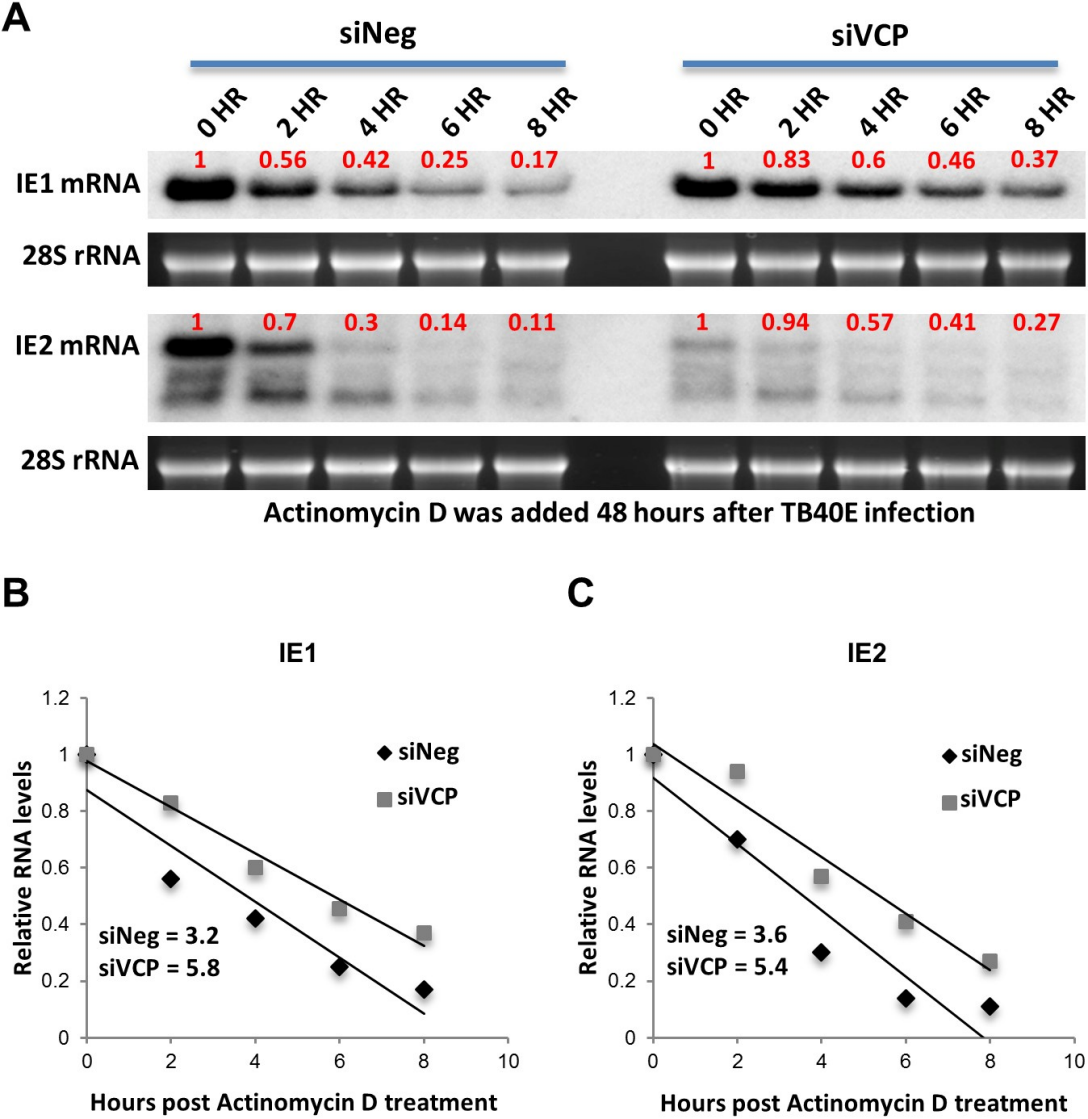
Knockdown of VCP does not reduce IE2 transcript stability. (A) Fibroblast cells were transfected with siRNA against VCP or negative control siRNA and infected with HCMV 48 hours post transfection. Forty-eight HPI cells were treated with actinomycin D to inhibit transcription and total RNA harvested at the indicated time points. Stability of IE1 and IE2 transcript was determined by Northern blot analysis. Bands were quantified using Image J software [54]. Quantification of transcript levels shown in (A) were plotted and transcript half-life determined using equations derived from linear trend lines. Knockdown of VCP results in an increase in transcript stability of 1.8 fold for IE1 (5.8 for VCP knockdown cells versus 3.2 for control cells) (B) and 1.5 fold for IE2 (5.4 for VCP knockdown versus 3.6 for control cells) (C).

To determine whether loss of VCP results in specific destabilization of IE2 transcript, RNA levels were measured following treatment of cells with actinomycin D 48 HPI. Actinomycin D inhibits RNA polymerase, blocking new transcription of IE1 and IE2, allowing for monitoring of transcript stability over time. Total RNA was harvested at the indicated time points and IE1 and IE2 transcript levels determined by Northern blot analysis. Calculation of the half-life of IE1 and IE2 transcripts indicated that stability of both IE1 and IE2 modestly increased by 1.8 and 1.5 fold respectively, suggesting loss of VCP does not result in IE2 transcript destabilization (Fig 5).

### Knockdown of VCP alters splicing of MIE transcripts

**Fig 6:**
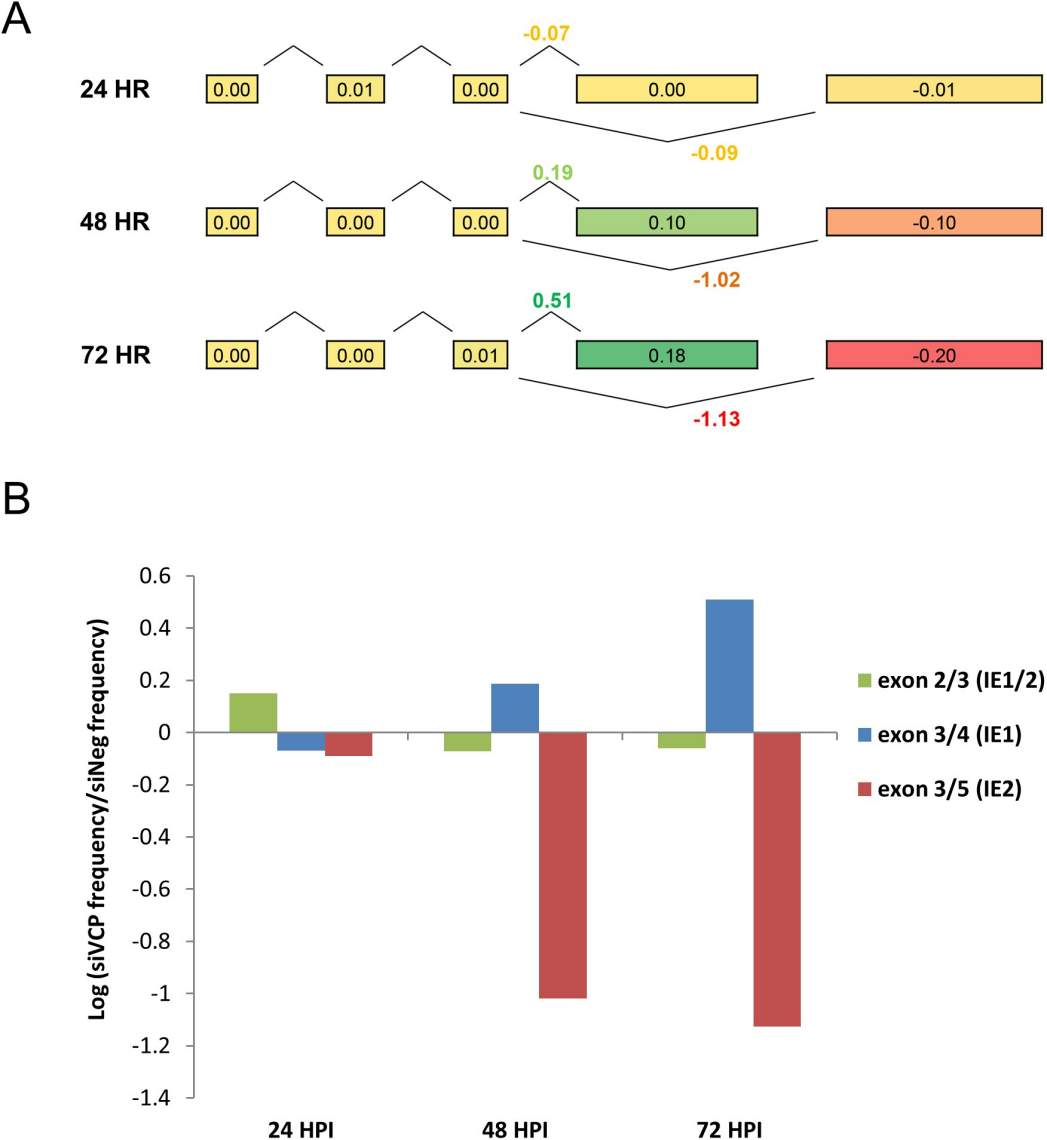
Knockdown of VCP alters splicing of MIE transcripts. RNA-seq analysis was performed to measure relative splicing levels of the MIE region following VCP knockdown. (A) The proportion of total reads mapping to each of the five exons was calculated, with the absolute difference in these values between VCP knockdown and corresponding negative control shown (numbers within exons). Knockdown of VCP has no effect on the proportion of reads originating from exons 1, 2 or 3, but is associated with a greater proportion of reads derived from exon 4 (IE1) and decreased numbers from exon 5 (IE2). (B) Log_2_ ratios of the proportions of reads mapping to exon 3 whose matching pair maps to either exons 2, 4 or 5 are shown. The proportion of read pairs spanning the shared splice junction between exon two and three is unaffected by VCP knockdown, whereas exon 3 to 4 frequency increases and 3 to 5 decreases.

Defining changes in alternative splicing of IE1 and IE2 is challenging as multiple factors, including promoter activity, RNA stability and viral genome amplification can all contribute to an increase or decrease in total IE1 and IE2 mRNA levels. To determine whether knockdown of VCP altered differential splicing of the MIE region, RNAseq analysis was performed. This allows a direct comparison of read counts from the first three shared exons of the MIE transcripts to the IE1 and IE2 specific exons four and five, respectively. This controls for changes in promoter activity and effects of genome amplification. Therefore, based on our current understanding of RNA processing, changes in the absolute ratio of exon four or five to the shared exons must be due to changes in splicing or changes in RNA stability. Cells were transfected with VCP siRNA or a negative control siRNA and infected with HCMV at an MOI of three. Total RNA was harvested at 24, 48 and 72 HPI and subjected to strand-specific, paired-end, Illumina sequencing. The annotated exons of the major immediate early region were clearly apparent from the mapped reads in all six samples and reads corresponding to the splice junctions were consistent with previous data for the region, indicating splicing between shared exons one to three and splicing between exon three and four, resulting in IE1 transcript, and three and five resulting in IE2 transcript (S3 Fig). Consistent with the Northern blot and qRT-PCR data, MIE transcription increased at 24 HPI but decreased at 48 and 72 HPI following knockdown of VCP (S4 Fig). Furthermore, splicing of the MIE primary transcript is heavily biased towards IE1 at 24 hours, with IE2 splicing increasing as the infection progresses (S4A Fig). To determine whether knockdown of VCP alters the balance of splicing between IE1 and IE2, the proportion of total reads mapping to the five exons of the IE region were calculated (S2 Table). Fig 6A represents the absolute difference in exon frequencies from VCP knockdown samples compared to negative control samples. At 24 HPI, knockdown of VCP has no effect on the relative proportion of reads mapping to exon four and five. However, by 48 HPI there is a clear increase in the relative proportion of reads mapping to exon four in VCP knockdown samples, with a corresponding decrease in exon five read frequencies. In contrast knockdown of VCP has no effect on the relative proportion of reads mapping to the shared exons, indicating reduced splicing from exon three to five (IE2) and a corresponding increase in splicing from exon three to four (IE1). Knockdown of VCP also resulted in lower frequencies of read pairs spanning exon three to five splice junction (IE2) and a corresponding increase in read pairs spanning exon three to four splice junction (IE1) (Fig 6A and 6B, S4B Fig and S3 and S4 Tables). These results show that knockdown of VCP alters splicing of MIE transcripts resulting in a failure to switch from IE1 splicing to IE2 splicing. To determine whether knockdown of VCP resulted in more generalized effects on virus transcript splicing, we analysed read counts for other well-characterized HCMV spliced transcripts (S5 Fig). Based on exon counts, only UL37 showed a similar pattern to MIE transcripts in response to VCP knockdown where absolute exon frequencies for UL37 exon 1 increased with a corresponding decrease in exon 3 read frequencies. However, unlike the MIE transcripts, analysis of paired-end reads did not support substantial splicing between UL37 exon 1 to exons 2 and 3 (S3 Fig). Instead, exon 1 and exons 2 and 3 of UL37 are likely to be predominantly independent transcriptional units with differing kinetics, with exon 2 and 3 requiring IE2 expression for transactivation.

### Expression of a subset of viral genes remains high following VCP knockdown, despite loss of IE2 expression

To determine the effect of VCP knockdown on global viral gene expression, read counts were mapped to the entire viral genome. As shown in Supplemental Fig 6 total read counts mapping to the viral genome were relatively similar at 24 HPI. However, while viral gene expression increased in control cells by approximately 6.5 fold between 24 and 72 HPI, expression in VCP knockdown cells only increased by 3-fold, indicating a general reduction in viral gene expression following VCP knockdown. This is unsurprising given the vital role IE2 protein plays in transactivation of viral gene expression.

**Fig 7:**
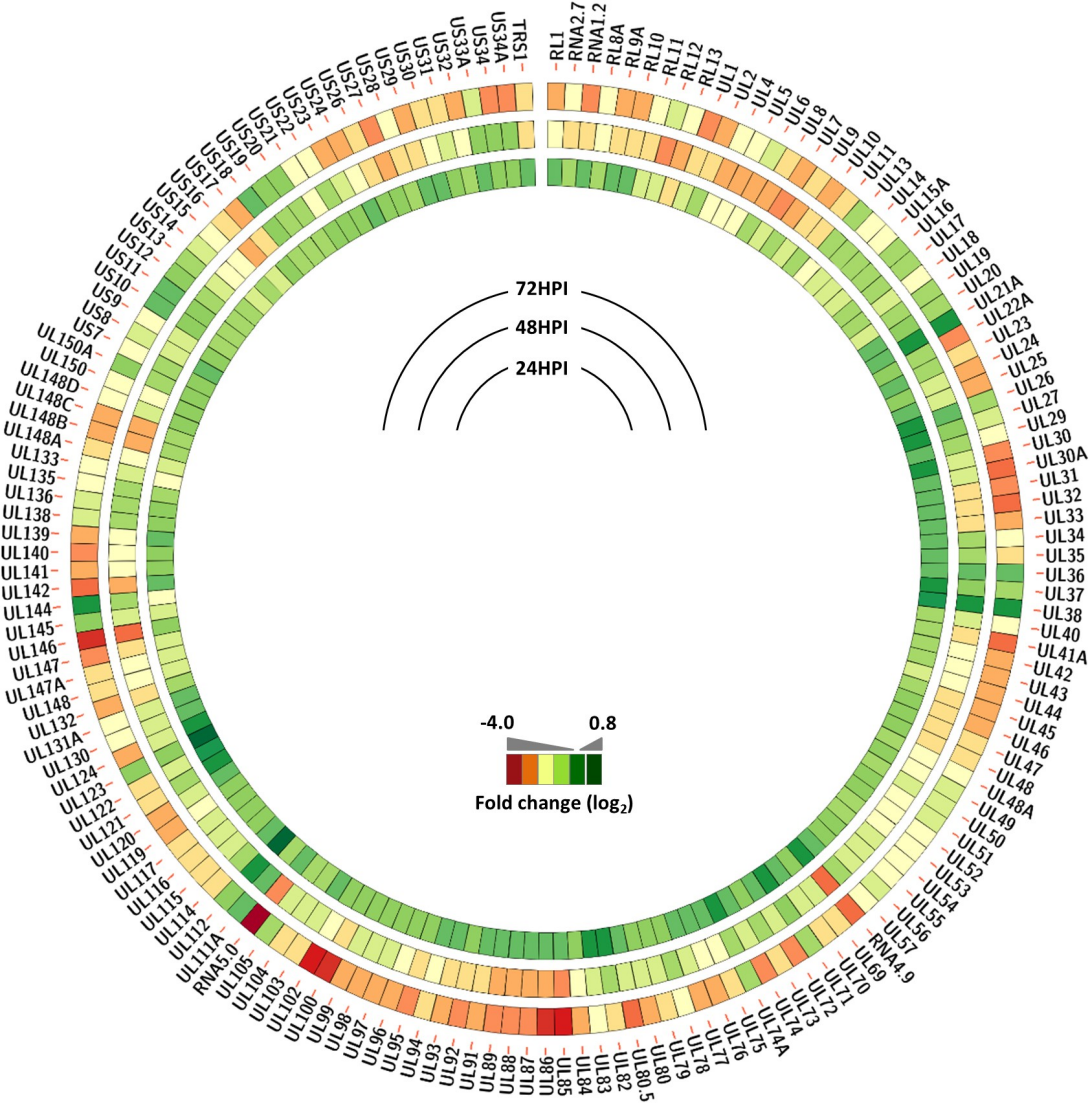
Expression of a subset of viral genes remains high following VCP knockdown, despite loss of IE2 expression. Read counts were aligned to the TB40E genome sequence and normalised for total reads and CDS length. Difference in transcription at each time point was determined by calculating the Log_2_ ratio in normalised read counts between control infected cells and VCP knockdown infected cells.

To determine whether knockdown of VCP results in a similar effect on all viral genes or differential effects, total reads were mapped to individual viral open reading frames and viral gene expression compared between control cells and VCP knockdown cells (Fig 7 and S5 Table). At 24 HPI moderate reductions in viral gene expression can be observed, with a subset of transcripts expressed at relatively higher levels following VCP knockdown. In particular, expression levels of UL36 and UL37, UL112/113 and the MIE transcripts UL122 (IE2) and UL123 (IE1) were relatively higher at 24HPI in VCP knockdown cells compared to control cells. However, at 48 and 72 HPI there is more profound global suppression of virus gene expression in VCP knockdown cells. This is particularly apparent for the non-coding genes RNA 4.9 and 5.0 and other genes that are highly up regulated at later stages of infection, including UL85, UL86, UL100 and UL102. Despite the loss of IE2 expression, the expression of a subset of viral genes remained equivalent to control levels following knockdown of VCP (Fig 7 and S7 Fig). These include UL21A, UL36 and UL38, UL111A and UL112/113, UL123 (IE1) and UL144. This is particularly surprising for UL112/113 which has previously been identified as a target for IE2 transactivation [31,32].

### Altered MIE splicing is not due to changes in cell cycle or delay in replication progression following VCP knockdown

**Fig 8:**
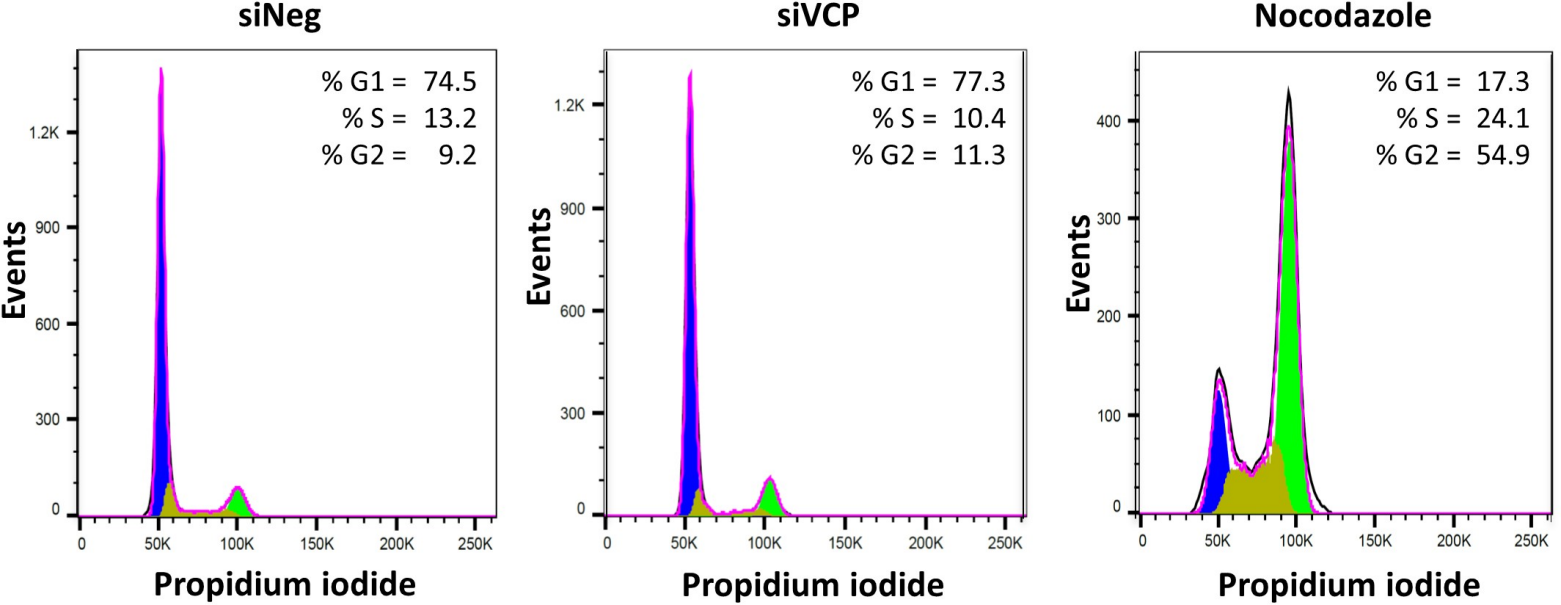
Altered MIE splicing is not due to changes in cell cycle following VCP knockdown. Fibroblast cells were transfected with negative control siRNA or VCP siRNA and cells were fixed and stained with propidium iodide 4 days post transfection. Untransfected cells were treated with 0.5 μg/ml nocodazole as a positive control of G2/M arrest. DNA content was then measured by FACS analysis and defined as G1, S or G2 using FlowJo software.

Previous studies have shown that the cell cycle impacts on the regulation of MIE alternative splicing with IE2 splicing and virus replication blocked at the G2/M phase [33]. Furthermore, cyclin dependent kinases are required for efficient IE2 expression and cyclin A2 overexpression inhibits IE2 splicing and virus replication [34]. To determine whether knockdown of VCP results in alterations in cell cycle, which in turn regulates MIE splicing, cells were stained with propidium iodide following VCP knockdown. The results clearly show that knockdown of VCP has no gross effect on cell cycle control (Fig 8). Consistent with the majority of cells being in G0/G1 phase, cyclin A2 levels remained undetectable by western blot in control and VCP knockdown cells (S8 Fig). These results show that regulation of MIE splicing by VCP is not a consequence of alterations in cell cycle control and instead represents a novel regulatory pathway. To determine whether the effect of VCP on MIE expression could be the result of a simple delay or inhibition of progression in virus replication, we compared MIE protein and transcript levels following treatment of cells with Ganciclovir, a well characterized drug, which inhibits viral DNA synthesis. While both Ganciclovir treatment and VCP knockdown result in substantial reductions in IE2 gene expression, only knockdown of VCP results in a corresponding increase in IE1 gene expression and relative changes in IE1 and IE2 splicing (S9 Fig). Furthermore, in contrast to VCP knockdown, IE2 and pp52 expression could be detected four days post infection following Ganciclovir treatment. These data suggest alterations in MIE levels, caused by VCP knockdown are not an artifact of delayed virus replication.

### VCP colocalises with viral replication compartments in the nucleus

**Fig 9:**
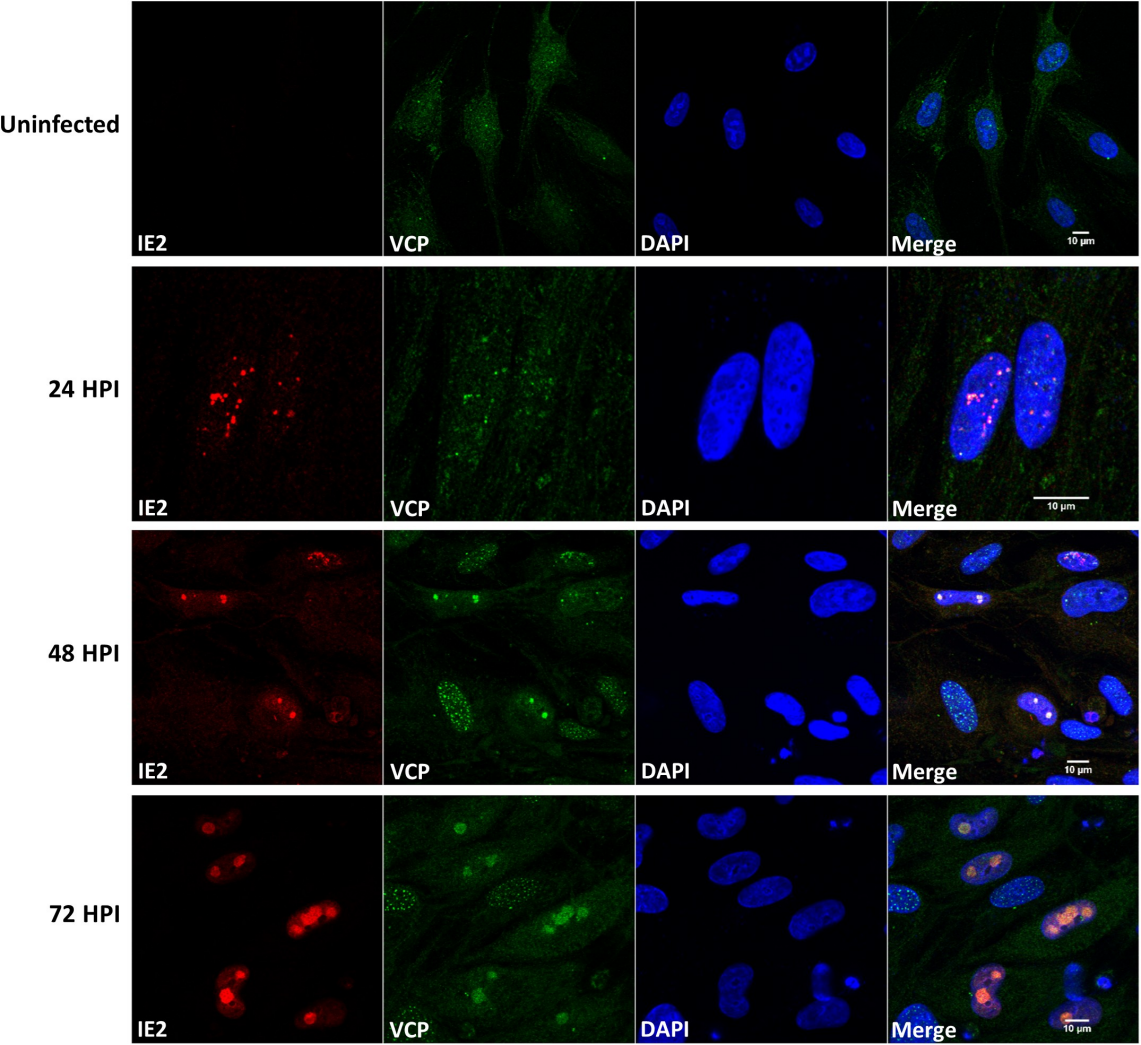
VCP colocalises with viral replication compartments in the nucleus. The cellular localisation of VCP was determined by confocal microscopy during HCMV infection. Cells were infected at high MOI and fixed at 24, 48 and 72 HPI and co-stained for VCP and IE2. Nuclei are stained with DAPI.

Previous studies have demonstrated that VCP plays an important role in the cytoplasm of HCMV infected cells, mediating US11-dependent degradation of MHC class I, by stripping the protein from the ER membrane in a ubiquitin dependent manner [27]. To determine the cellular localisation of VCP during HCMV infection, primary fibroblast cells were infected at high MOI with HCMV, fixed at 24-hour time points and stained for VCP. As shown in Fig 9, VCP displayed dynamic temporal cellular localisation during HCMV infection. In uninfected cells, VCP staining resulted in diffuse signal throughout the cell. However following infection with HCMV, distinct puncta can be observed in the nucleus of infected cells. By 48 HPI VCP was consistently found within two large puncta within the nucleus, which increased in size by 72 hours. Such staining is characteristic of the viral replication compartments. Following infection with HCMV the viral genomes are deposited adjacent to ND10 domains before forming two distinct replication compartments within the nucleus, characterized by colocalisation of viral DNA, DNA replication proteins and IE2 protein [35]. Therefore, VCP localisation to the virus replication compartments in the nucleus coincides with increased IE2 expression.

### VCP inhibitor NMS-873 is a potent inhibitor of HCMV replication

**Fig 10:**
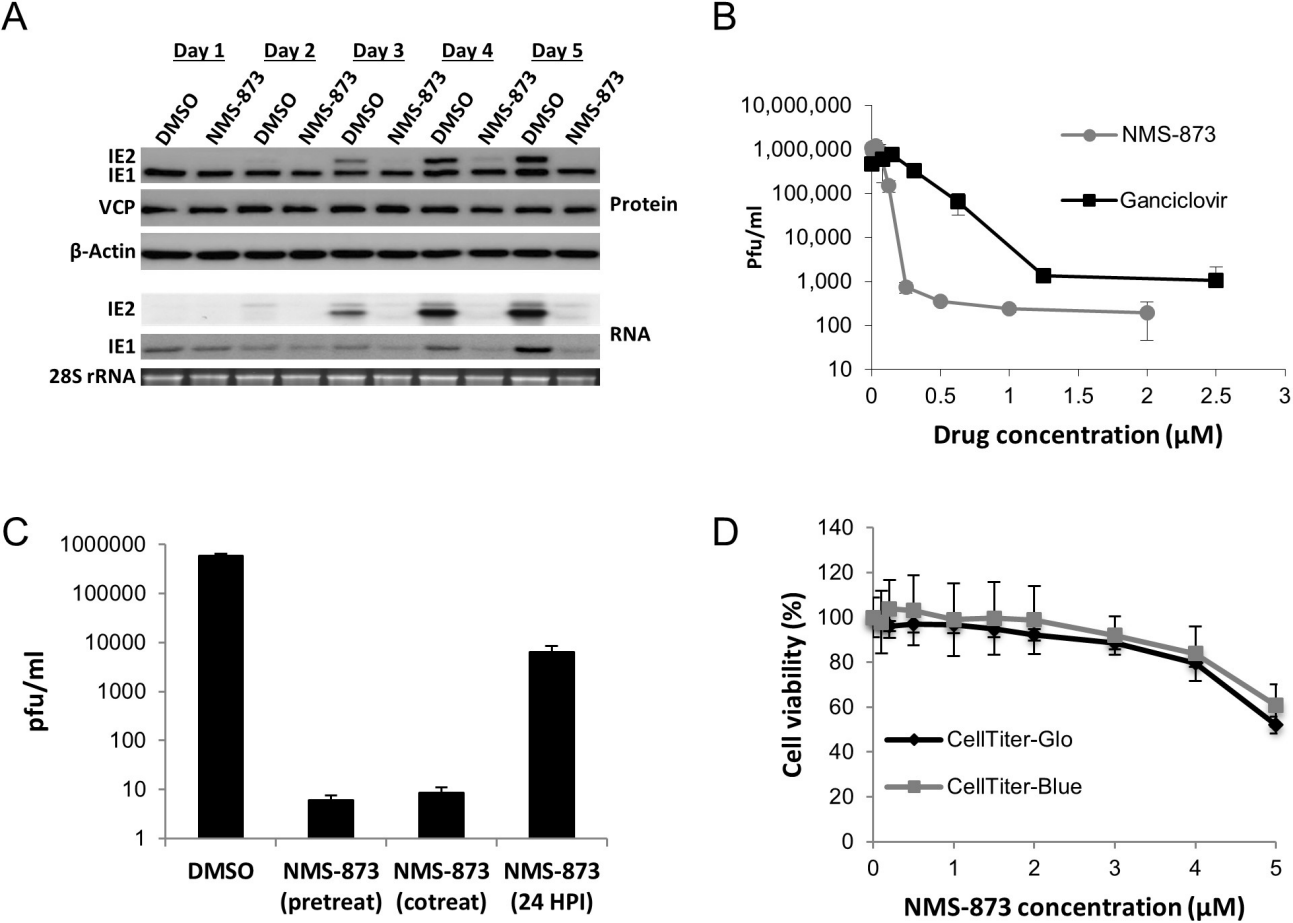
VCP inhibitor NMS-873 is a potent HCMV antiviral. (A) Treatment with NMS-873 causes the same phenotype as knockdown of VCP. Cells were pretreated with 1μM NMS-873 then infected at high MOI with HCMV. Total RNA and protein were harvested at the indicated times and IE1 and IE2 protein and RNA levels measured by Western and Northern blot analysis. (B) Cells were treated with the indicated concentrations of NMS-873 or Ganciclovir then infected 24 hours post treatment. Cell and supernatant was harvested at the indicated time points and virus levels determined by plaque analysis. (C) Cells were treated with NMS-873 24 hours before infection, at the same time as infection, or 24 HPI. Supernatant was collected seven days post infection and virus titers determined by plaque assay. (D) Cell viability was determined five days post treatment at the indicated concentrations of NMS-873 using two independent commercial kits, Cell Titer-Glo and Cell Titer-Blue. (B-D) Data represents two biological repeats.

Given that VCP is essential for HCMV replication, we investigated whether small molecule inhibitors against VCP are viable antiviral candidates. Because VCP is a potential anti-cancer target, a number of effective small molecule inhibitors of VCP have been developed. NMS-873 is a highly potent and selective inhibitor of VCP [36]. To determine whether treatment of infected cells with NMS-873 results in the same phenotype as VCP siRNA knockdown, cells were pretreated with NMS-873 or DMSO and infected at high MOI with HCMV. Total protein was harvested every 24 hours for a total of five days and subjected to western blot analysis. As shown in Fig 10A, treatment with 1μM of NMS-873 recapitulated the phenotypic effects of VCP siRNA, with loss of IE2 transcript and protein expression. In contrast to siRNA knockdown of VCP, treatment with NMS-873 did not result in increased IE1 expression. This may be due to additional off target effects of the drug compared to siRNA knockdown or a consequence of inhibiting the activity of VCP compared to directly knocking down the protein. Next, we compared the antiviral activity of NMS-873 to Ganciclovir, currently the most commonly used HCMV antiviral. Primary human fibroblast cells were pretreated with different concentrations of Ganciclovir, NMS-873 or DMSO control, with cells and supernatant harvested seven days post infection for plaque assay. As shown in Fig 10B, NMS-873 and Ganciclovir clearly inhibited the production of infectious virus. However, NMS-873 displayed a higher level of potency at ten-fold lower concentrations, with an IC50 of 0.13 μM compared to 1.3 μM for Ganciclovir. Co-treating cells with NMS-873 at the same time as infection also resulted in reduced IE2 levels and a similar reduction in infectious virus (Fig 10C and S10A Fig). In contrast, treating cells 24 HPI did not reduce IE2 expression or virus production to the same extent (Fig 10C and S10B Fig). While immediate early and early viral gene expression was not substantially affected by treatment of cells with NMS-873 at 24 hour post infection, expression of the late gene pp28 was substantially reduced, with infectious virus reduced 100 fold, suggesting VCP may also be involved in late processes of virus replication. This is consistent with the pleiotropic nature of VCP and its potential to affect HCMV replication in a multitude of ways. Treatment with NMS-873 also showed little toxicity at effective concentrations (Fig 10D), suggesting NMS-873 and small molecule inhibitors of VCP show significant potential as HCMV antiviral therapies.

## Discussion

VCP plays a pivotal role in ubiquitin-dependent signaling through remodeling target proteins, often leading to proteosomal degradation. As such it is linked to a wide range of biological functions, including protein quality control, autophagy, chromatin remodeling and more recently RNA processing [21]. Following systematic screening of host genes involved in membrane organization, we identified VCP as a critical host factor for HCMV replication. Further characterization demonstrated that knockdown of VCP resulted in an initial increase in MIE transcription, followed by a substantial reduction in the expression of the major immediate early transcript IE2. Strikingly, despite being expressed from the same primary transcript, IE1 levels increased following VCP knockdown. Our data also suggests that VCP plays a role during late stages of virus replication as inhibition of VCP 24 HPI did not substantially affect IE2 expression but still resulted in a two-log reduction in infectious virus. Given the functionally pleotropic nature of VCP, it is unsurprising that knockdown results in multifactorial effects on the virus and potentially reflects the diverse roles of ubiquitin modulation and signaling during virus infection.

Viral gene expression during HCMV replication occurs through a tightly regulated cascade mechanism with expression of the MIE genes IE1 and, in particular, IE2, required for transactivation of early viral genes and subsequent late viral gene expression. Due to the central role of the MIE proteins in driving replication of the virus they have been suggested to play a pivotal role in regulating the balance between productive and latent infection. IE1 and IE2 are generated from the same primary transcript by alternative splicing, sharing the first three exons, while splicing to independent final exons through the relatively unusual process of terminal exon skipping [10,12]. Consistent with previous publications [33,34], our data shows that IE1 expression levels increase more rapidly than IE2, due to preferential splicing to the IE1 terminal exon 4, resulting in lower levels of IE2 transcription, which plateaus between 12 and 24 HPI (S2, S3 and S4 Figs). As infection progresses, splicing to IE2 terminal exon 5 increases, with a corresponding decrease in IE1 splicing. Regulation of alternative splicing of the MIE transcripts is therefore crucial for determining whether replication of the virus progresses, as expression of IE2 is dependent on this switch in splicing. Although the timing and precise splicing pattern of the MIE region has been well documented, how the splicing is regulated and the downstream consequences for virus replication are poorly understood [10]. However, it is likely to depend on a complex interplay of cellular and viral factors and the architecture of the MIE transcriptional region [37].

Expression of the MIE transcripts and their splicing are intimately linked to multiple factors including cell cycle, cell type and differentiation status [13,38,39]. Suppression of MIE transcripts during HCMV infection of myeloid cultures is thought to be key for establishing latent infection. Conversely, activation and differentiation of latently infected cells results in up-regulation of MIE gene expression and subsequent reactivation. During infection of fibroblast cells, expression of MIE transcripts and replication are closely linked to cell cycle status, with MIE expression suppressed during S/G2 phase [40,41]. This suppression is due in part to cyclin A2, which alters splicing of the MIE transcripts in a manner similar to knockdown of VCP, resulting in loss of IE2 expression and failure in virus replication [33]. While expression of cyclin A2 results in a similar phenotype, our data shows that VCP knockdown does not cause gross changes in the cell cycle or increased cyclin A2 expression, indicating an alternative mechanism of action.

Although the majority of research on VCP has focused on its cytoplasmic activity, recent studies have identified equally important and diverse roles for VCP in the nucleus. The association of VCP with the viral replication compartments early in infection suggests that the nuclear functions of VCP may be playing a critical role in HCMV replication. Studies in yeast and mammalian cell lines have demonstrated that VCP modulates the association of factors with chromatin, leading to regulation of cell cycle, transcription, DNA replication and DNA damage response, all of which could potentially affect virus replication and MIE expression [21]. Previous reports have shown that HCMV induces the host DNA damage response, with response factors including ATM, γH2AX and E2F1 required for efficient HCMV replication and IE2 expression [42]. γH2AX also colocalises with the viral replication compartments in the nucleus. VCP acts downstream of ATM and is required for the removal of ubiquitinated factors associated with dsDNA break regions, allowing repair to proceed [21]. If knockdown of VCP disrupts the DNA damage response during HCMV infection, this could contribute to the observed loss of IE2 expression and inhibition of virus replication.

Reports are also emerging that VCP plays a role in transcript stability and regulation of splicing. HuR is a RNA binding protein that stabilizes transcripts by binding to canonical AU rich regions within 3’UTRs. Following ubiquitination of HuR in response to stress signals, VCP removes HuR from associated transcripts resulting in transcript destabilization [43]. In Drosophila, VCP has been linked to dendrite pruning in neuronal cells [44]. Expression of mutant forms of VCP results in incorrect splicing of a gene important to dendritic pruning, MICAL, possibly through mis-localisation of the RNA binding protein TDP-43. Given the known mechanism of action of VCP it is likely that any effects on MIE splicing are indirect, occurring through targeting of a ubiquitinated intermediate protein. One possible model is that a negative regulator of IE2 splicing exists at early stages during HCMV infection, which promotes IE1 expression at the expense of IE2 expression. Based on the transcriptional architecture of the major immediate region, such a factor could regulate MIE splicing by promoting polyadenylation of the IE1 transcript, thereby inhibiting read-through to the IE2 final exon. The factor would be ubiquitinated and removed by VCP between 12 and 24 HPI, allowing read-through of the first polyadenylation site and splicing of the IE2 transcript. This would be consistent with the initial plateau in IE2 transcription and localisation of VCP to the replication compartments at 12 to 24 HPI, where spliceosome and proteasome components have also been reported to colocalise [30,45]. The NMS-873 data also supports this model, with inhibition of IE2 expression only effective with either pre-treatment or addition of the drug at the same time as infection. Treatment at 24 HPI was ineffective at blocking IE2 expression, suggesting a window in which VCP activity is required for regulation of MIE splicing. Interestingly, blocking *de novo* protein translation by cycloheximide treatment, effectively rescues IE2 transcription following NMS-873 treatment, suggesting the inhibitory factor is induced by virus infection (S11 Fig).

Although not common, there are a number of examples of terminal exon skipping in the human genome, which would be analogous to the proposed model. Maturation of B cells to differentiated plasma cells results in a switch from predominantly membrane bound IgM to secreted IgM. Studies have shown that increased expression of the polyadenylation factor cstF-64 promotes polyadenylation upstream of the exons required for membrane bound IgM resulting in increased expression of the secreted form [46,47]. Tissue specific regulation of calcitonin peptide is also regulated through differential polyadenylation of an embedded terminal exon [48]. Here, the splicing factor SRSF3 (SRp20) binds to a polyA enhancer sequence and directs polyadenylation of the upstream polyA site through recruitment of polyA factors. Restricting SRSF3 activity results in removal of the upstream terminal exon by splicing and enhanced expression of the downstream terminal exon. In these examples splicing is directly linked to cellular differentiation, in the case of IgM, and tissue specificity, in the case of calcitonin. These factors are fundamental to the regulation of HCMV latency with cell type specificity determining the site of latency and differentiation of latently infected cells linked to reactivation [13,14]. Given the central role of IE2 in determining virus replication, linking regulation of MIE splicing to cellular differentiation and tissue specificity would be a potent mechanism of regulating the establishment, maintenance and reactivation of latency in HCMV. Experiments are currently underway to determine whether regulation of MIE splicing by VCP follows a similar paradigm to cellular examples and whether such regulation is involved in HCMV latency.

Finally, as VCP is clearly essential for virus replication, small molecule inhibitors of VCP are potentially attractive antiviral candidates. HCMV-related illness accounts for more than 60% of diseases associated with solid organ transplant patients. Prolonged treatment, especially in patients with severely suppressed immune systems, greatly increases the risk of antiviral resistance [49–51]. Very few antivirals have been developed for use against HCMV since the licensing of Ganciclovir, and of these, the same viral genes are targeted, reducing the likely usefulness of these drugs against resistant strains [52]. An alternative strategy for the development of novel antivirals involves targeting of host genes required by the virus for successful replication. VCP has been identified as a potential anti-cancer target and as such a number of small molecule inhibitors have been developed, the most potent of which is NMS-873 [36]. We show that NMS-873 is 10 times more potent than Ganciclovir at equivalent concentrations, with little sign of toxicity at active levels. Whether the drug is toxic *in vivo* remains to be determined. Deletion of VCP in transgenic mice is non-viable and naturally occurring mutations in humans are linked to severe developmental defects [21,53]. However, transient inhibition of VCP by small molecule inhibitors may be a viable treatment option, especially in patients where resistance to current antivirals poses a significant risk to health.

## Materials and methods

### Cell culture and viral infection

Normal Human Dermal Fibroblasts (NHDF; Gibco) were maintained in Dulbecco’s modified high glucose media (DMEM; Sigma) supplemented with 10% fetal bovine serum (FBS; Gibco) and 1% penicillin-streptomycin (Invitrogen). A low passage HCMV strain TB40E, which expresses GFP from an SV40 promoter was used for siRNA library screening, western blot analysis, and northern blot analysis. Laboratory adapted HCMV strain AD169 was used for immunofluorescence experiments.

### siRNA library screening and transfection

The Human siGENOME siRNA Library that targets 140 membrane trafficking genes (4 siRNAs per gene; Dharmacon, Inc.) and 20 other selected genes were included in the primary screen. In brief, NHDFs were seeded in 96-well plate a day before siRNA transfection. Next day, cells reached 90–95% confluency and were transfected with siRNA twice (4 hours apart between first and second transfections) using Lipofectamine RNAiMAX (Invitrogen) according to the manufacturer’s protocol. Transfected NHDFs were incubated for 48 hours and then infected with GFP expressing TB40E virus at an MOI of three. GFP intensity was monitored every 24 hours with Synergy HT microplate reader (Biotek). The entire screen was performed in duplicate and repeated three times. VCP gene identified from the primary screen was subsequently silenced with four individual siRNAs to eliminate the possibility that the phenotype was associated with off target effects.

### Western blot analysis

Cells were lysed with RIPA buffer (0.1% SDS, 1% Triton X-100, 1% deoxycholate, 5 mM EDTA, 150 mM NaCl, and 10 mM Tris at pH 7.2) containing protease inhibitor cocktail (Roche). Ten micrograms of the total lysate was separated in 10% SDS-polyacrylamide gels and transferred to PVDF membranes (Millipore). Primary antibodies used in this paper are mouse anti-CMV IE1/2 monoclonal antibody (MAB8131, Millipore), mouse anti-CMV pp52 monoclonal antibody (CH16, Santa Cruz Biotechnology), mouse anti-CMV pp28 monoclonal antibody (CH19, Santa Cruz Biotechnology), rabbit anti-VCP polyclonal antibody (H-120, Santa Cruz Biotechnology), and mouse anti-β-Actin monoclonal antibody (Abcam). Blots were probed with primary antibody (1:500–1:2000) diluted in 5% dehydrated milk in Tris Buffered Saline (TBS) and subsequently the HRP-conjugated secondary antibodies (Pierce) at 1:5000. Blots were washed in TBS three times, incubated with chemiluminescent substrate (SuperSignal West Pico; Thermo Scientific) according to the manufacturer’s protocol, and exposed in G:Box (Syngene) for visualization of bands.

### Northern blot analysis

Total RNA was isolated by using Trizol solution according to the manufacturer’s protocol. Northern blot analysis for IE1 and IE2 mRNAs was conducted using total RNA that was separated on a 1.2% agarose formaldehyde gel and then transferred using Whatman TurboBlotter Rapid Downward Transfer Systems. IE1 and IE2 probes were generated by PCR using cDNA from TB40E infected NHDFs (as template) and labelled with Amersham Rediprime II DNA labelling system (GE Healthcare) with the following primers (IE1: TCAAACAGATTAAGGTTCGAGTGG, and ATCCACATCTCCCGCTTATCCTCG; IE2: TCATGGTGCGCATCTTCTCCACC, and TTACTGAGACTTGTTCCTCAGGTCC). After hybridization and wash, the membranes were exposed to autoradiography film with an intensifying screen (Biomax Transcreen HE, Kodak) for visualization of bands.

### RNAseq analysis

Cells were transfected and infected as described above. Total RNA was harvested using Trizol according to manufacturers guidelines. Total RNA was submitted to Edinburgh Genomics for generation of TruSeq stranded libraries and subjected to HiSeq high output v4 125PE sequencing. The strand-specific RNA-seq reads were mapped to a combined version of the human (hg38) and human herpesvirus 5 (KF297339) genomes using the HISAT spliced read mapper (PMID:25751142). Only valid read pairs mapped together in the correct orientation were retained (i.e. carrying a SAM flag of one of 83, 99, 147 or 163) and a custom Perl script was used to count the number of these reads mapping to each exon as well as the number of read pairs spanning different combinations of exons.

### Drug inhibitor studies and cytotoxicity assay

NMS-873 (Xcess Biosciences) and Ganciclovir (Cambridge Bioscience) were dissolved in DMSO and added to the cell culture at a working concentration 24 hours before HCMV infection. Viability for NMS-873 treated cells was assessed using the CellTiter-Glo luminescent cell viability assay kit (Promega) and CellTiter-Blue (Promega) 120 hours after NMS-873 addition. MG132 (Cambridge Bioscience), Pepstatin A (Sigma), and E64 (Sigma) were dissolved in DMSO and added to the cell culture 24 hours after HCMV infection. 100 μg/ml cycloheximide (in DMSO, Sigma) was added to the cell culture 30 minutes before HCMV infection to block protein biosynthesis.

### Immunofluorescence

Laboratory adapted HCMV strain AD169 infected cells were fixed and permeabilized in Methanol:Acetone solution (1:1) for 7 minutes, and then blocked with 5% human serum in PBS for 30 minutes. Primary and secondary antibodies were diluted with 5% human serum in PBS. Cells were washed with PBS after primary and after secondary antibody incubations. Primary antibodies used in this paper are mouse anti-CMV IE2 monoclonal antibody (12E2, Santa Cruz Biotechnology), rabbit anti-VCP polyclonal antibody (H-120, Santa Cruz Biotechnology) at 1:500. Alexa-fluor-647 conjugated goat anti-mouse or Alexa-fluor-488 conjugated goat-anti-rabbit IgG secondary antibodies were diluted 1:1000. All images were acquired with Zeiss LSM 710 confocal microscope fitted with 63X/1.4 oil-immersion objective lens.

### Real-time PCR analysis

Total RNA was isolated by using Trizol solution according to the manufacturer’s protocol followed by DNase (Turbo DNA-free kit, Ambion) treatment, and then reverse transcribed with poly T primers using High Capacity cDNA Reverse Transcription Kit (Invitrogen). Real-Time PCR was carried out using by Taqman assays with pre-designed gene-specific primer/probe set (Applied Biosystems) on Rotor gene 3000 (Corbet Research). Custom primer/probe set are CGTCAAACAGATTAAGGTTCGAGTGG, CCACATCTCCCGCTTATCCTCG, and 56-FAM/CATGCTCTG/ZEN/CATAGTTAGCCCAATACACTTCATCTC- CTCG/3IABkFQ for IE1, and ATGGTGCGCATCTTCTCCACC, TTACTGAGACTTGTTCCTCAGGTCCTG, and 56-FAM/CAGGCTCAG/ZEN/GGTGTCCAGGTCTTCGGGAGG/3IABkFQ for IE2.

### Flow cytometry cell cycle analysis

Cells were harvested with trypsin, washed with PBS, and followed by fix with 70% cold ethanol for 30 minutes at 4°C. Then the fixed cells were washed twice with PBS, treated with RNaseA, and labelled with 10 μl of propidium iodide (1 mg/ml stock solution, Sigma). Cell cycle analysis was first carried out using BD LSRFortessa X20, and then further analyzed using FlowJo software.

## Supporting information

S1 Fig Cell viability was determined following transfection of negative control siRNA versus VCP siRNA five days post transfection, using two different commercial kits, CellTiter Glo and CellTiter Blu. (DOCX)

S2 Fig Total RNA was generated as above with IE1 and IE2 levels determined by quantitative RT-PCR. Expression was normalised to GAPDH with relative levels of IE1 and IE2 compared to the time point showing highest expression. Comparison of relative IE1 and IE2 levels in negative control cells (A) and VCP knockdown cells (B). Comparison between NEG and VCP knockdown cells of relative IE1 (C) and IE2 (D) expression levels. Error bars represent standard deviation from two biological repeats.
(DOCX)

S3 Fig Total read counts mapping to the MIE and UL37 region of HCMV. Sashimi plots of the exon and splice junction coverage across the MIE and UL37 genes at different time points and VCP levels (knockdown or control). Read depth on the gene’s corresponding strand are indicated with bar graphs. Reads spanning splice junctions are represented by arcs, with counts indicating the number of reads split across the corresponding junction. All numbers representing un-normalised raw read counts. Low frequency, background splicing events were filtered out in both plots (MIE: minimum splice count of 20, UL37 minimum of 15).
(DOCX)

S4 Fig Relative share of read counts aligning to exon 4 (IE1) or Exon 5 (IE2). Read counts were normalised for CDS length and reads per million (Fragments per kilobase million–FPKM). Total normalised read counts aligning to exons four and five at 24, 48 and 72 HPI in control cells (A) or VCP knockdown cells (B).
(DOCX)

S5 Fig Knockdown of VCP does not cause general defect in viral transcript splicing. The proportion of total reads mapping to exons of known HCMV spliced transcripts was calculated, with the absolute difference in these values between VCP knockdown and corresponding negative control shown (numbers within exons).
(DOCX)

S6 Fig Normalised read counts mapping to KF297339 viral genome (TB40E). The total number of normalised total read counts were mapped to open reading frames of TB40E to determine the effects of VCP knockdown on global viral transcription.
(DOCX)

S7 Fig Expression of a subset of viral genes remains high despite VCP knockdown and loss of IE2 expression. (DOCX)

S8 Fig Fibroblast cells were transfected with negative control or VCP siRNA and total protein harvested at the indicated time points. Cyclin A2 levels were determined by Western blot analysis. HEK293 lysate was included as a positive control for cyclin A2 detection.
(DOCX)

S9 Fig Effects on MIE splicing is not due to block in progression of virus replication. (A) Western blot from Fig 2B showing virus protein levels following VCP knockdown (B) Virus protein expression following inhibition of virus replication with ganciclovir. (C) Quantification of difference in IE1 protein levels between knockdown of VCP versus ganciclovir treatment. Quantification is compared to negative control for each time point. (D) Relative IE1 and IE2 transcript levels normalised to MIE shared exons. Levels were determined by qRT-PCR using primer probes specific to exon 1 to 3, exon 4 or exon 5. Exon 4 and 5 levels were then normalised to exon 1–3 for VCP knockdown cells (D) or cells treated with Ganciclovir (5 μM).
(DOCX)

S10 Fig IE2 expression not substantially blocked when NMS-873 is added 24 hours post infection. Western blot analysis of immediate early (IE1 and IE2), early (pp52) and late (pp28) gene expression following treatment of cells at the same time as infection (A) or 24 hours post infection (B) with 1 μM NMS-873.
(DOCX)

S11 Fig Cycloheximide rescues IE2 RNA expression following NMS-873 treatment. Cells were treated with DMSO or NMS-873 24 hours prior to infection at high MOI with HCMV. Cells were treated 100 μg/ml cycloheximide, 30 minutes prior to infection to block *de novo* protein synthesis and total RNA harvested at indicated times. IE1 and IE2 transcript levels were determined by Northern blot analysis.
(DOCX)

S1 Table siRNA screen data. Raw data from the three repeated siRNA screens including technical repeats. Average over the three experiments as well as Z scores and standard deviations are shown.
(XLSX)

S2 Table Exon read count ratios. Total read counts for each of the five exons for each condition are shown along with ratio to total calculations and differential between negative control and VCP knockdown samples.
(XLSX)

S3 Table Splice junction counts. Total read counts across each of the 3 MIE junctions are shown along with ratio calculations defining the differential between exon 2 to 3, 3 to 4 and 3 to 5 in log 2.
(XLSX)

S4 Table Relative expression levels of exons 4 and 5 are significantly dependent on the combination of timepoint and VCP status. Linear model results of testing associations between the relative expression levels of each exon and both time and siVCP treatment. Relative expression levels being measured as the proportion of the transcript’s reads that mapped to the corresponding exon in that sample. Coefficients, standard errors (in brackets) and p values are shown. Associations between relative expression levels of exons 4 and 5 and timepoint are dependent on VCP status as demonstrated by the significant interaction term (Time:siVCP).
(DOCX)

S5 Table Global analysis of viral gene expression following VCP knockdown. Read counts (FPKM) aligning to open reading frames of TB40E at 24, 48 and 72 HPI in control cells or VCP knockdown cells. Log_2_ ratios of read counts between VCP knockdown cells and control cells are shown.
(XLSX)
