# Supplementary material for: The host ubiquitin-dependent segregase VCP/p97 is required for the onset of human cytomegalovirus replication
Source: PLoS Pathog. 2017 May 11;13(5):e1006329. doi: 10.1371/journal.ppat.1006329 (PMC5426786; doi:10.1371/journal.ppat.1006329)
Supplement: S1 Fig — (DOCX) [file ppat.1006329.s001.docx]

**Supplemental Figure 1.** Cell viability was determined following transfection of negative control siRNA versus VCP siRNA five days post transfection, using two different commercial kits, CellTiter Glo and CellTiter Blu.
