# Supplementary material for: The host ubiquitin-dependent segregase VCP/p97 is required for the onset of human cytomegalovirus replication
Source: PLoS Pathog. 2017 May 11;13(5):e1006329. doi: 10.1371/journal.ppat.1006329 (PMC5426786; doi:10.1371/journal.ppat.1006329)
Supplement: S2 Fig — Expression was normalised to GAPDH with relative levels of IE1 and IE2 compared to the time point showing highest expression. Comparison of relative IE1 and IE2 levels in negative control cells (A) and VCP knockdown cells (B). Comparison between NEG and VCP knockdown cells of relative IE1 (C) and IE2 (D) expression levels. Error bars represent standard deviation from two biological repeats. (DOCX) [file ppat.1006329.s002.docx]

**Supplemental Figure 2.** Total RNA was generated as above with IE1 and IE2 levels determined by quantitative RT-PCR. Expression was normalized to GAPDH with relative levels of IE1 and IE2 compared to the time point showing highest expression. Comparison of relative IE1 and IE2 levels in negative control cells (A) and VCP knockdown cells (B). Comparison between NEG and VCP knockdown cells of relative IE1 (C) and IE2 (D) expression levels. Error bars represent standard deviation from two biological repeats.
