# Supplementary material for: The host ubiquitin-dependent segregase VCP/p97 is required for the onset of human cytomegalovirus replication
Source: PLoS Pathog. 2017 May 11;13(5):e1006329. doi: 10.1371/journal.ppat.1006329 (PMC5426786; doi:10.1371/journal.ppat.1006329)
Supplement: S3 Fig — Sashimi plots of the exon and splice junction coverage across the MIE and UL37 genes at different time points and VCP levels (knockdown or control). Read depth on the gene’s corresponding strand are indicated with bar graphs. Reads spanning splice junctions are represented by arcs, with counts indicating the number of reads split across the corresponding junction. All numbers representing un-normalised raw read counts. Low frequency, background splicing events were filtered out in both plots (MIE: minimum splice count of 20, UL37 minimum of 15). (DOCX) [file ppat.1006329.s003.docx]

**Supplemental Figure 3. Total read counts mapping to the MIE and UL37 region of HCMV.** Sashimi plots of the exon and splice junction coverage across the MIE and UL37 genes at different time points and VCP levels (knockdown or control). Read depth on the gene’s corresponding strand are indicated with bar graphs. Reads spanning splice junctions are represented by arcs, with counts indicating the number of reads split across the corresponding junction. All numbers representing un-normalised raw read counts. Low frequency, background splicing events were filtered out in both plots (MIE: minimum splice count of 20, UL37 minimum of 15).


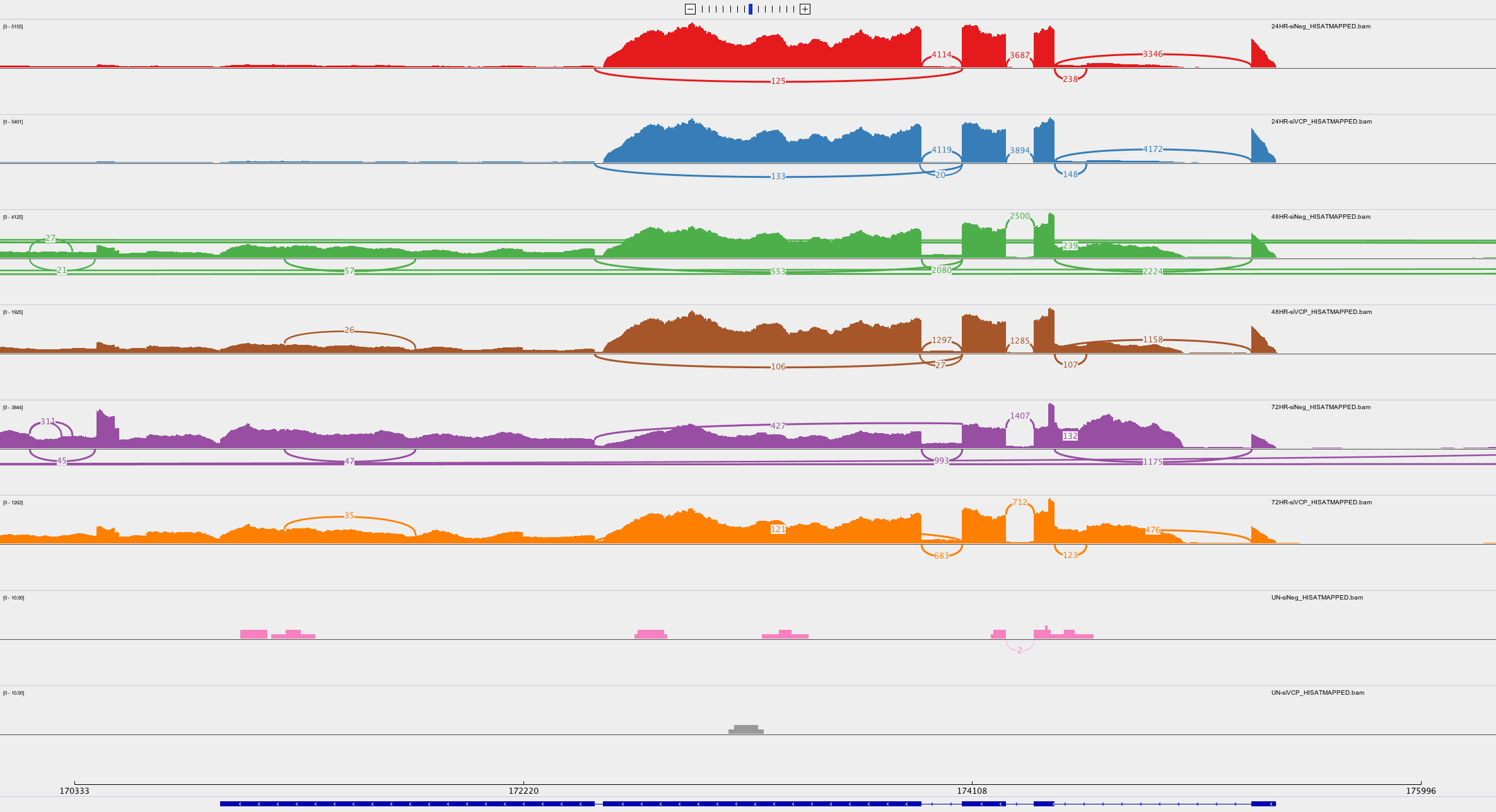


24 Hours siNEG

24 Hours siVCP

48 Hours siNEG

48 Hours siVCP

72 Hours siNEG

72 Hours siVCP

x5 (IE2)

4x (IE1)

x3

x2

x1


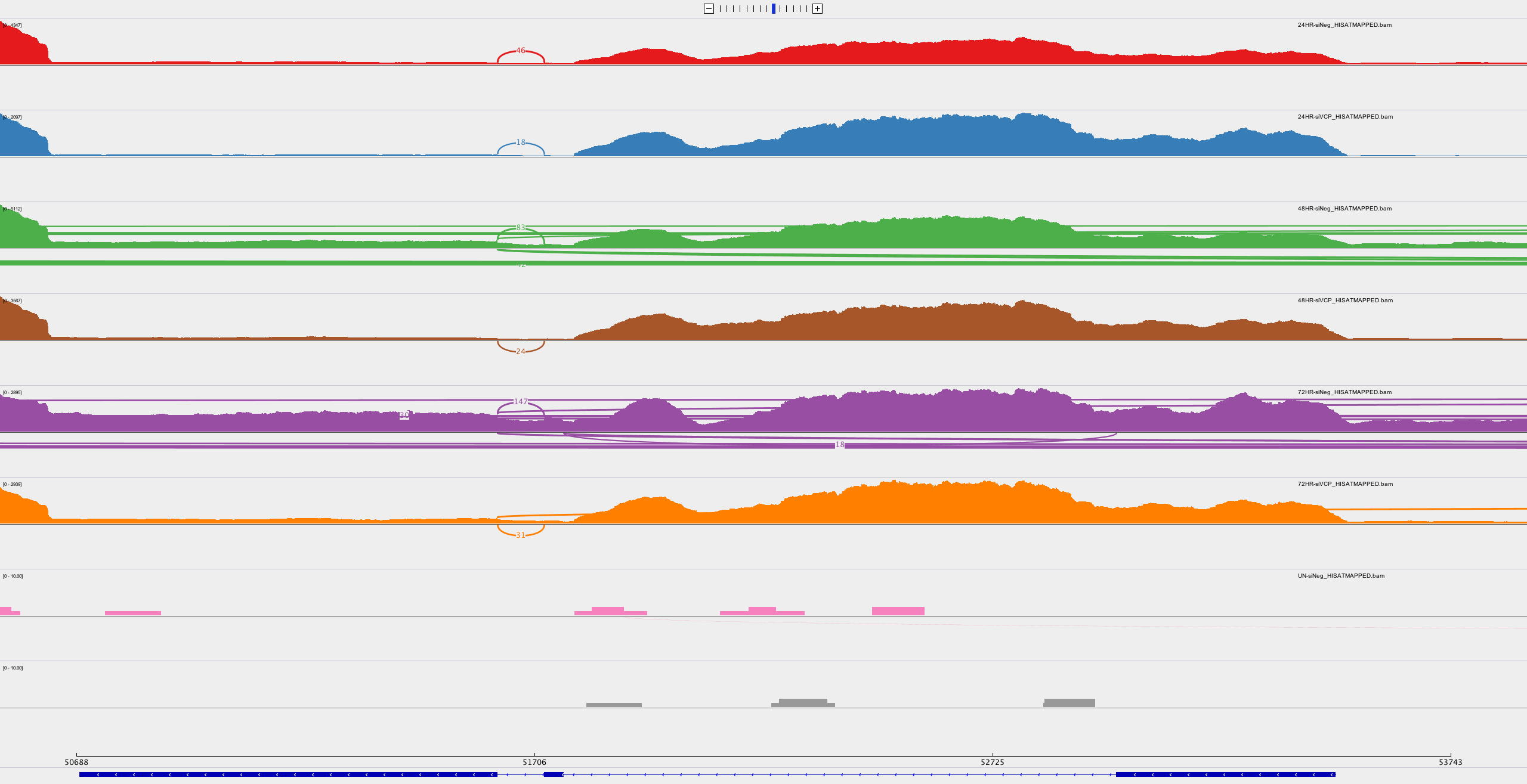


UL37x1

UL37x2

UL37x3

UL38

24 Hours siNEG

24 Hours siVCP

48 Hours siNEG

48 Hours siVCP

72 Hours siNEG

72 Hours siVCP
