# Supplementary material for: The host ubiquitin-dependent segregase VCP/p97 is required for the onset of human cytomegalovirus replication
Source: PLoS Pathog. 2017 May 11;13(5):e1006329. doi: 10.1371/journal.ppat.1006329 (PMC5426786; doi:10.1371/journal.ppat.1006329)
Supplement: S4 Fig — Read counts were normalised for CDS length and reads per million (Fragments per kilobase million–FPKM). Total normalised read counts aligning to exons four and five at 24, 48 and 72 HPI in control cells (A) or VCP knockdown cells (B). (DOCX) [file ppat.1006329.s004.docx]

**Supplemental Figure 4. Relative share of read counts aligning to exon 4 (IE1) or Exon 5 (IE2).** Read counts were normalised for CDS length and reads per million (Fragments per kilobase million – FPKM). Total normalised read counts aligning to exons four and five at 24, 48 and 72 HPI in control cells (A) or VCP knockdown cells (B).
