# Supplementary material for: The host ubiquitin-dependent segregase VCP/p97 is required for the onset of human cytomegalovirus replication
Source: PLoS Pathog. 2017 May 11;13(5):e1006329. doi: 10.1371/journal.ppat.1006329 (PMC5426786; doi:10.1371/journal.ppat.1006329)
Supplement: S5 Fig — The proportion of total reads mapping to exons of known HCMV spliced transcripts was calculated, with the absolute difference in these values between VCP knockdown and corresponding negative control shown (numbers within exons). (DOCX) [file ppat.1006329.s005.docx]

**Supplemental Figure 5. Knockdown of VCP does not cause general defect in viral transcript splicing.** The proportion of total reads mapping to exons of known HCMV spliced transcripts was calculated, with the absolute difference in these values between VCP knockdown and corresponding negative control shown (numbers within exons).
