# Supplementary material for: The host ubiquitin-dependent segregase VCP/p97 is required for the onset of human cytomegalovirus replication
Source: PLoS Pathog. 2017 May 11;13(5):e1006329. doi: 10.1371/journal.ppat.1006329 (PMC5426786; doi:10.1371/journal.ppat.1006329)
Supplement: S6 Fig — The total number of normalised total read counts were mapped to open reading frames of TB40E to determine the effects of VCP knockdown on global viral transcription. (DOCX) [file ppat.1006329.s006.docx]

**Supplemental Figure 6. Normalised read counts mapping to KF297339 viral genome (TB40E).** The total number of normalised total read counts were mapped to open reading frames of TB40E to determine the affects of VCP knockdown on global viral transcription.
