# Supplementary material for: The host ubiquitin-dependent segregase VCP/p97 is required for the onset of human cytomegalovirus replication
Source: PLoS Pathog. 2017 May 11;13(5):e1006329. doi: 10.1371/journal.ppat.1006329 (PMC5426786; doi:10.1371/journal.ppat.1006329)
Supplement: S7 Fig — (DOCX) [file ppat.1006329.s007.docx]

**Supplemental Figure 7. Expression of a subset of viral genes remains high despite VCP knockdown and loss of IE2 expression.**
