# Supplementary material for: The host ubiquitin-dependent segregase VCP/p97 is required for the onset of human cytomegalovirus replication
Source: PLoS Pathog. 2017 May 11;13(5):e1006329. doi: 10.1371/journal.ppat.1006329 (PMC5426786; doi:10.1371/journal.ppat.1006329)
Supplement: S8 Fig — Cyclin A2 levels were determined by Western blot analysis. HEK293 lysate was included as a positive control for cyclin A2 detection. (DOCX) [file ppat.1006329.s008.docx]

**Supplemental Figure 8.** Fibroblast cells were transfected with negative control or VCP siRNA and total protein harvest at the indicated time points. Cyclin A2 levels were determined by Western blot analysis. HEK293 lysate was included as a positive control for cyclin A2 detection.
