# Supplementary material for: The host ubiquitin-dependent segregase VCP/p97 is required for the onset of human cytomegalovirus replication
Source: PLoS Pathog. 2017 May 11;13(5):e1006329. doi: 10.1371/journal.ppat.1006329 (PMC5426786; doi:10.1371/journal.ppat.1006329)
Supplement: S9 Fig — (A) Western blot from Fig 2B showing virus protein levels following VCP knockdown (B) Virus protein expression following inhibition of virus replication with ganciclovir. (C) Quantification of difference in IE1 protein levels between knockdown of VCP versus ganciclovir treatment. Quantification is compared to negative control for each time point. (D) Relative IE1 and IE2 transcript levels normalised to MIE shared exons. Levels were determined by qRT-PCR using primer probes specific to exon 1 to 3, exon 4 or exon 5. Exon 4 and 5 levels were then normalised to exon 1–3 for VCP knockdown cells (D) or cells treated with Ganciclovir (5 μM). (DOCX) [file ppat.1006329.s009.docx]

**Supplemental Figure 9.** Effects on MIE splicing is not due to block in progression of virus replication. (A) Western blot from figure 2B showing virus protein levels following VCP knockdown (B) Virus protein expression following inhibition of virus replication with ganciclovir. (C) Quantification of difference in IE1 protein levels between knockdown of VCP versus ganciclovir treatment. Quantification is compared to negative control for each time point. (D) Relative IE1 and IE2 transcript levels normalised to MIE shared exons. Levels were determined by qRT-PCR using primer prbes specific to exon 1 to 3, exon 4 or exon 5. Exon 4 and 5 levels were then normalised to exon 1-3 for VCP knockdown cells (E) or cells treated with Ganciclovir (5μM).
