# Supplementary material for: The host ubiquitin-dependent segregase VCP/p97 is required for the onset of human cytomegalovirus replication
Source: PLoS Pathog. 2017 May 11;13(5):e1006329. doi: 10.1371/journal.ppat.1006329 (PMC5426786; doi:10.1371/journal.ppat.1006329)
Supplement: S10 Fig — Western blot analysis of immediate early (IE1 and IE2), early (pp52) and late (pp28) gene expression following treatment of cells at the same time as infection (A) or 24 hours post infection (B) with 1 μM NMS-873. (DOCX) [file ppat.1006329.s010.docx]

**Supplemental Figure 10. IE2 expression not substantially blocked when NMS-873 is added 24 hours post infection.** Western blot analysis of immediate early (IE1 and IE2), early (pp52) and late (pp28) gene expression following treatment of cells at the same time as infection (A) or 24 hours post infection (B) with 1μM NMS-873.

**(A)**

**(B)**
