# Supplementary material for: The host ubiquitin-dependent segregase VCP/p97 is required for the onset of human cytomegalovirus replication
Source: PLoS Pathog. 2017 May 11;13(5):e1006329. doi: 10.1371/journal.ppat.1006329 (PMC5426786; doi:10.1371/journal.ppat.1006329)
Supplement: S11 Fig — Cells were treated with DMSO or NMS-873 24 hours prior to infection at high MOI with HCMV. Cells were treated 100 μg/ml cycloheximide, 30 minutes prior to infection to block de novo protein synthesis and total RNA harvested at indicated times. IE1 and IE2 transcript levels were determined by Northern blot analysis. (DOCX) [file ppat.1006329.s011.docx]

**Supplemental Figure 11. Cycloheximide rescues IE2 RNA expression following NMS-873 treatment.** Cells were treated with DMSO or NMS-873 24 hours prior to infection at high MOI with HCMV. Cells were treated 100μg/ml cycloheximide, 30 minutes prior to infection to block *de novo* protein synthesis and total RNA harvested at indicated times. IE1 and IE2 transcript levels were determined by Northern blot analysis.
