# Supplementary material for: The host ubiquitin-dependent segregase VCP/p97 is required for the onset of human cytomegalovirus replication
Source: PLoS Pathog. 2017 May 11;13(5):e1006329. doi: 10.1371/journal.ppat.1006329 (PMC5426786; doi:10.1371/journal.ppat.1006329)

**Supplemental Table 4. Relative expression levels of exons 4 and 5 are significantly dependent on the combination of timepoint and VCP status**. Linear model results of testing associations between the relative expression levels of each exon and both time and siVCP treatment. Relative expression levels being measured as the proportion of the transcript’s reads that mapped to the corresponding exon in that sample. Coefficients, standard errors (in brackets) and p values are shown. Associations between relative expression levels of exons 4 and 5 and timepoint are dependent on VCP status as demonstrated by the significant interaction term (Time:siVCP).


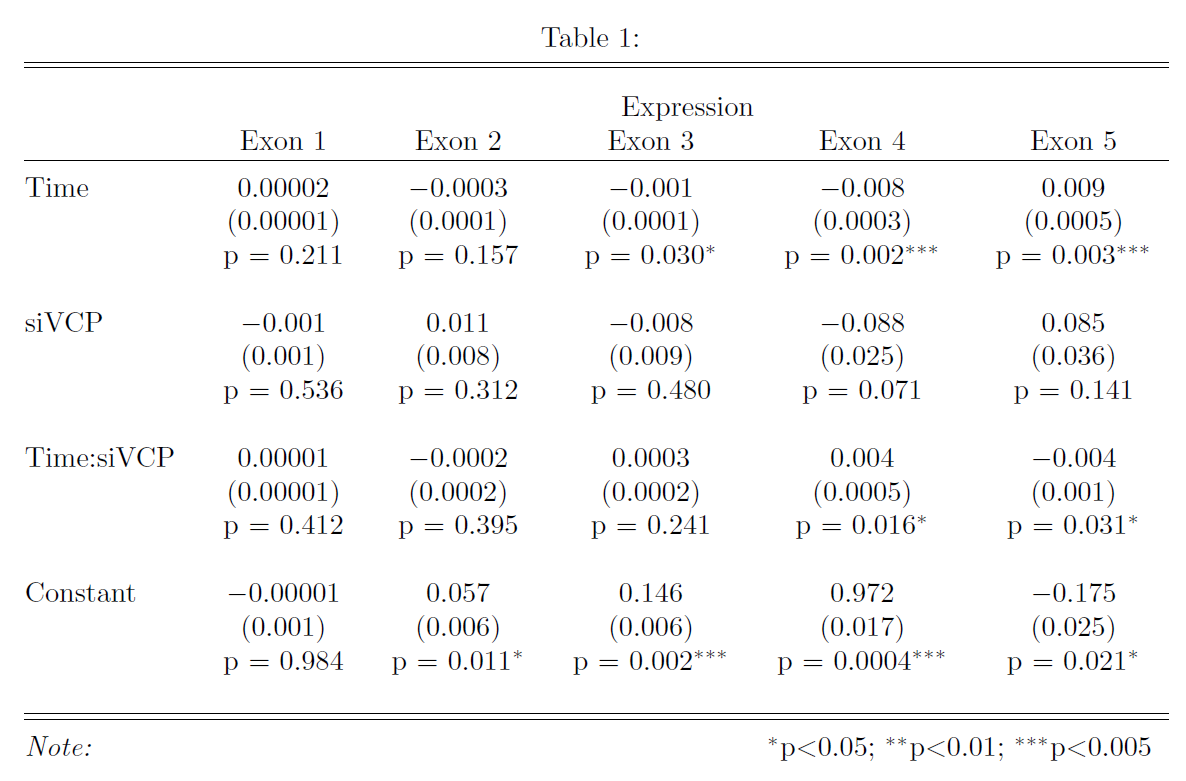

Supplement: S4 Table — Linear model results of testing associations between the relative expression levels of each exon and both time and siVCP treatment. Relative expression levels being measured as the proportion of the transcript’s reads that mapped to the corresponding exon in that sample. Coefficients, standard errors (in brackets) and p values are shown. Associations between relative expression levels of exons 4 and 5 and timepoint are dependent on VCP status as demonstrated by the significant interaction term (Time:siVCP). (DOCX) [file ppat.1006329.s015.docx]
